# Recent Advances in Barnacle-Inspired Biomaterials in the Field of Biomedical Research

**DOI:** 10.3390/ma18030502

**Published:** 2025-01-22

**Authors:** Tiantian Min, Zhongna Zhang, Lan Chen, Jingan Li

**Affiliations:** School of Materials Science and Engineering, Zhengzhou University, Zhengzhou 450001, China; mintiantian@stu.zzu.edu.cn (T.M.); zhongna_zhang@gs.zzu.edu.cn (Z.Z.)

**Keywords:** barnacle cement protein, barnacle adhesion curing mechanism, biomedical materials, barnacle-inspired research

## Abstract

As a marine fouling organism, barnacles secrete a cement whose proteins self-assemble into stable nanofibers, conferring exceptional underwater adhesion and curing properties. The barnacle cement proteins (BCPs) are of significant interest in biomedicine due to their adhesiveness, water resistance, stability, and biocompatibility, making them ideal for developing novel biomaterials. Additionally, BCPs have wound-healing acceleration and antibacterial properties, offering new insights for antimicrobial biomaterial development. Recently, barnacle-inspired materials have seen extensive research and notable progress in biomedicine. As the understanding of barnacle cement and its adhesion mechanisms deepens, their medical applications are expected to expand. This review summarizes the latest advancements of barnacle biomimetic materials in biomedicine, including their use in adhesives, tissue engineering, drug delivery, and hemostasis, highlighting their characteristics, applications, and potential research directions, and providing a comprehensive reference for the field.

## 1. Introduction

In the marine environment, barnacles secrete a highly adhesive and cross-linkable underwater barnacle cement [1,2], which firmly attaches to various hard surfaces in the ocean and is difficult to remove. The large-scale attachment of barnacles causes damage to marine infrastructure, such as seawater pipes, hulls, and dock piles, while also affecting the balance of the surrounding marine ecological environment, severely impacting people’s production and life. Early research focused mainly on the separation, purification, and physicochemical property analysis of barnacle cement proteins. With the advancement of science and technology, especially the development of molecular biology and nanotechnology, research on barnacle biomimetic materials has gradually penetrated to the molecular level and nanoscale, laying a solid foundation for their application in the biomedical field.

Barnacle cement is composed of highly hydrophobic and glycosylated proteins [3], rapidly solidifying and demonstrating strong adhesion in the underwater environment through protein cross-linking and self-assembly [4]. Barnacle cement, made up of multi-protein complexes [5], is favored in biomedical field research due to its strong adhesive force, solidification characteristics, excellent waterproofness, high stability, non-toxicity, and good biocompatibility, making it an ideal choice for new functional bio-medical materials. Barnacle glue proteins not only accelerate wound healing but also possess antimicrobial functions and mineralization properties, providing new avenues for the development of multifunctional biomedical materials [6,7,8,9]. Therefore, further research on barnacle cement not only helps us to thoroughly understand the attachment and solidification mechanisms of marine organisms, but also provides important insights for the design and development of biomimetic hemostatic adhesives [2,10,11], soft tissue substitutes [12], bone regeneration barrier membranes [13,14], drug delivery systems [15,16], antimicrobial medical materials [8], and other biomimetic medical materials. In addition, barnacle cement may contain components that inhibit bacterial growth and promote biodegradation [17,18], providing new ideas for the development of antimicrobial bio-medical materials.

Barnacle biomimetic medical materials are a class of materials developed and inspired by the characteristics of barnacles, revealing the unique adhesion and curing mechanisms exhibited by barnacles during the process of biological evolution. These materials provide a theoretical basis for the design and development of new medical materials, and have achieved a certain level of progress. The research on barnacle biomimetic materials in the biomedical field is not only of great significance but also has a broad application prospect in production practice, making a significant contribution to the development of bio-medical materials.

## 2. The Basic Properties of Barnacle Cement

### 2.1. Specific Types and Functions of Proteins in Barnacle Cement

Barnacle cement is a complex biological adhesive composed of multiple proteins [19,20] that can rapidly solidify under aquatic conditions, forming a strong adhesive force that allows barnacles to firmly attach to various underwater surfaces. A variety of proteins have been isolated from barnacle cement, including Mrcp-19k [21], Mrcp-20k [21], Mrcp-52k [22], Mrcp-68k [5], Mrcp-100k [5], Balcp-19k [23], and Balcp-20k [24]. These proteins have different molecular weights and functions, and they exert their adhesive functions by self-assembling into nanofibers and adsorbing specific substances under various conditions.

Mrcp19k is an important adhesive protein from the red giant barnacle (*Megabalanus rosa*). Through genetic engineering methods, various fusion proteins have been constructed by fusing different protein functional modules with Mrcp19k. The fusion adhesive proteins, such as Mrcp19k-Ddx4 and the reticulated adhesive protein CC-Mrcp19k, have shown significantly enhanced adhesive and elastic properties in underwater adhesion tests [25,26], demonstrating better underwater adhesion and non-aqueous adhesion performance than non-fusion adhesive proteins.

Mrcp20K is another primary adhesive protein from the red giant barnacle, which has been efficiently expressed in Pichia pastoris through in vitro genetic recombination techniques [27]. Studies have indicated that strategies such as increasing gene dosage, the co-expression of auxiliary proteins, and combining high-density fermentation can significantly enhance the expression of Mrcp20K to the gram level [28]. Furthermore, by fusing and expressing with Aspergillus oryzae lipase, the expression of Mrcp20K was further increased to 410 ± 10.00 mg/L [28]. Additionally, Mrcp20k protein has shown interactions with Ca^2+^ [29], which may contribute to its function in biomineralization processes, offering potential applications in biomimetic materials for bone tissue engineering.

Mrcp-52k is a hydrophobic protein with glycosylation modifications, obtained through heterologous expression systems such as Escherichia coli and Pichia pastoris. It forms a dense reticular fiber on material surfaces, facilitating protein adhesion. Additionally, Mrcp-52k plays a key role in the curing process of barnacle adhesion, potentially promoting further curing and stabilization of the glue through its self-assembly properties [26].

Mrcp-100k has leucine (Leu) as the most abundant amino acid residue, followed by serine (Ser) and isoleucine (Ile). The first 18 amino acid residues are considered signal peptides, and their high hydrophobicity suggests that Mrcp-100k is a secretory protein [5].

Regarding the specific structural characteristics of Mrcp-68, detailed descriptions were not found in the search; however, it can be inferred that it may possess characteristics similar to the aforementioned multi-protein complexes and may be involved in regulating the physical properties of the glue, such as adhesion and elasticity [26].

Balcp19k, a protein from the white ridge barnacle (*Balanus albicostatus*), self-aggregates under acidic and low ionic strength conditions to form a highly viscous glue, which is closely related to the adhesion and curing process of barnacles. Research indicates that recombinant Balcp-19k exhibits time-dependent secondary structural transitions and self-assembles into non-amyloid nanofibers under acidic and low ionic strength conditions [30]. These nanofibers are highly stable and do not disassemble in seawater. Their adhesiveness is stronger than that of unassembled protein monomers and can resist the adverse effects of high pH and high ionic strength. This suggests that the self-assembly of barnacle glue proteins into non-amyloid fibers has a significant impact on curing, enhancing the stability and adhesion of materials. Moreover, this self-assembly process does not require the cooperation of other bio-adhesive proteins or auxiliary components. Based on the aforementioned characteristics of Balcp-19k, it offers potential for the research and development of biomimetic materials for medical use, such as hemostatic dressings and adhesives, the surface modification of implanted metal stents, and bone tissue engineering [31]. Bal-cp20k protein triggers self-assembly behavior in response to pH changes, forming water-insoluble precipitates, which may be a result of self-assembly [32,33].

In summary, the proteins in barnacle cement are primarily Mrcp-19k, Mrcp-20k, Mrcp-52k, Mrcp-68, Mrcp-100k, Balcp-19k, Balcp-20k, etc., which have been engineered through various genetic techniques to enhance their adhesive performance and elastic capabilities in both moist and non-moist environments.

### 2.2. Self-Assembly of Barnacle Cement Proteins

#### 2.2.1. The Self-Assembly Process of Barnacle Cement Proteins

The self-assembly process of barnacle cement proteins is a complex biochemical process that primarily relies on protein–protein interactions [34], especially the formation of disulfide bonds [5,35,36], to stabilize protein structures and adhesive capabilities. For instance, the cp20k protein can interact with other cement proteins such as cp19k under pH 8.0 conditions, an interaction that may be triggered by the transition from an acidic environment (such as within the cement gland) to the weakly alkaline marine environment [32]. Moreover, the disulfide bonds of the cp20k protein are crucial for its binding to Ca^2+^, which aids in its role in biomineralization processes [24,29]. Disulfide bonds are covalent bonds formed by the oxidation of thiol groups from two cysteine residues within proteins [23], possessing high bond energy (30–100 kcal/mol). These bonds can exist within the same polypeptide chain or between different chains, effectively linking different peptide chains or different parts of the same chain to stabilize the protein’s spatial structure.

In the self-assembly process of barnacle adhesive proteins, the formation of disulfide bonds involves the effective pairing of cysteine residues, oxidation reactions, and the potential catalytic action of Protein Disulfide Isomerase (PDI), which can be influenced by various factors. Firstly, the spatial arrangement and proximity of cysteine residues during protein folding and self-assembly are key to the formation of disulfide bonds. Studies have shown that disulfide bonds tend to form between sequences less than 70 amino acids apart and are more likely to appear in the first half of the amino acid sequence [23,37]. This suggests that specific amino acid sequences and spatial structures in barnacle adhesive proteins may facilitate the effective pairing and oxidation of cysteine residues, with the kinetics of disulfide bond formation affecting the rate of self-assembly of barnacle adhesive proteins. Research indicates that correct disulfide bond pairing is crucial for the proper folding of polypeptide chains, and incorrect pairing may lead to protein misfolding, affecting its function [5,24]. The formation of disulfide bonds is subject to the catalytic action of Protein Disulfide Isomerase (PDI) [36]. PDI is a molecular chaperone that not only catalyzes the formation of disulfide bonds but also rearranges incorrectly paired disulfide bonds [38,39]. This implies that during the self-assembly of barnacle adhesive proteins, PDI may help form stable disulfide bonds by promoting the correct pairing of cysteine residues and preventing incorrect pairing. In vitro experiments have shown that engineered methods designing protein–ligand pairs with specific disulfide bonds can enhance protein–protein interactions, thereby promoting the formation of disulfide bonds during self-assembly [40,41]. Additionally, disulfide bonds have a crucial impact on maintaining the active function of proteins. For instance, studies have found that when disulfide bonds are mutated or missing, the biological activity of proteins significantly decreases [36]. This indicates that during the self-assembly of barnacle adhesive proteins, disulfide bonds may maintain the activity of barnacle adhesive proteins in a dynamically changing form, ensuring their normal function. The cp20k protein can adsorb calcite, which may be related to its characteristics in disulfide bond formation [35,42]. This interaction not only helps barnacle adhesive proteins form stable structures in aquatic environments, but may also be involved in the biomineralization process of barnacle bases [43].

In the self-assembly process of barnacle adhesive proteins, interactions with other molecules, such as hydrogen bonds and electrostatic interactions, are also involved. For example, rBalcp19k primarily self-assembles through noncovalent interactions [23].

Furthermore, the self-assembly process of barnacle cement proteins is influenced by solution conditions, such as pH and ionic strength. Taking the cp19k protein as an example, it can self-assemble into non-amyloid nanofibers under acidic, low ionic strength conditions, and these special nanofibers are highly stable in seawater, effectively resisting the adverse effects of high pH and high ionic strength, thereby interfering with their self-assembly behavior [23,44]. This indicates that the self-assembly process of barnacle cement proteins depends not only on the protein’s own characteristics but is also closely related to external environmental conditions.

Overall, the self-assembly process of barnacle cement involves complex interactions between proteins, structural changes, and environmental factors, particularly the formation of disulfide bonds [35] to enhance protein stability and adhesive properties. This process is influenced by solution conditions such as pH and ionic strength and is closely related to the solidification of barnacle cement. These important findings provide significant molecular insights into the adhesion mechanism of barnacle cement and offer new inspiration for the development of novel biomimetic adhesives and other bio-medical biomimetic materials.

#### 2.2.2. Methods for Observing and Analyzing the Self-Assembly of Barnacle Cement Proteins

Observing and analyzing the self-assembly process of barnacle adhesive proteins can be achieved through various experimental methods, including infrared spectroscopy (IR), ultraviolet–visible spectroscopy (UV–Vis), turbidity measurement (TM), fluorescence spectroscopy (FS), circular dichroism (CD), nuclear magnetic resonance (NMR) spectroscopy, scanning electron microscopy (SEM), atomic force microscopy (AFM), transmission electron microscopy (TEM), and laser-scanning confocal microscopy (CLSM). The related detection methods and principles are summarized in Table 1.

In the process of protein self-assembly, significant changes occur in the amide I and amide II bands due to the formation of intermolecular hydrogen bonds and the alignment of peptide chains [45]. For instance, during self-assembly, protein molecules may transition from random coil structures to more ordered structures with an increased presence of β-sheets or α-helices. These conformational changes can be monitored through variations in the intensity and shape of the amide bands in Fourier Transform Infrared Spectroscopy (FTIR) spectra [46,47,48]. Additionally, FTIR can be employed to analyze potential hydration effects, solvent effects, and pH changes that may accompany the protein self-assembly process. These factors all influence the conformational stability of proteins, which are reflected in the infrared spectra. Thus, IR can be utilized to observe the self-assembly process of barnacle adhesive proteins. UV–Vis spectroscopy can be used to monitor changes in protein absorption spectra [49]; turbidity measurement can be employed to assess changes in solution turbidity, thereby indirectly reflecting the extent of protein self-assembly [46,50]; fluorescence spectroscopy is an effective method for detecting collagen self-assembly, including endogenous fluorescence and fluorescence probes, which are rapid, simple, accurate, and possess high sensitivity and selectivity [51]. The main fluorescence probes used for collagen currently include 3-methoxy-4′N,N-dimethylaminobenzyl (DMMF), pyrene fluorescence probes, and thioflavin T [52,53,54]. Circular dichroism is a non-destructive detection method that provides information on secondary structure composition without atomic-level detail [55], and N. J. Greenfield used this method to estimate protein secondary structure [56]. NMR spectroscopy is also a non-destructive detection method that offers information regarding collagen conformation changes, molecular motion, and binding interactions [17]. SEM allows for the observation of the surface and cross-sectional structure of self-assembled collagen-cellulose films with nanofibril layers [57]. Utilizing AFM rapid scanning imaging technology on an atomically flat mica substrate, researchers have successfully monitored the formation process of collagen fibrils and studied their self-assembly kinetics in real-time [58]. Nudelman et al. [59] used TEM to observe the mineralization process of hydroxyapatite (HAP) on collagen fibrils. CLSM can capture the process of protein self-assembly with the addition of fluorescent labels, allowing for the real-time observation of liquid–liquid phase separation phenomena and the morphology of the final self-assembled structures [60].

#### 2.2.3. Characteristics of Fiber Structures Formed by the Self-Assembly of Barnacle Cement Proteins

Barnacle cement proteins are capable of self-assembling into amyloid fibrils and non-amyloid nanofibers, with structural characteristics primarily reflected in their self-assembly behavior, microstructure, and interactions with environmental conditions. Proteins such as Balcp19k can self-assemble into amyloid fibrils in seawater solutions [23], which is closely related to the curing of barnacle cement, indicating that the self-assembly process is a key step in the solidification of barnacle cement. Additionally, rBalcp19k proteins exhibit time-dependent secondary structure transitions and self-assemble into non-amyloid nanofibers under acidic, low ionic strength conditions, which are very stable in seawater and do not disassemble [30].

Amyloid nanofibers are formed by the misfolding and aggregation of proteins, typically characterized by cross-β structure features [61]. This structure consists of two parallel-arranged polypeptide β-sheet layers, manifesting as highly ordered fibrous structures. The width of these fibers is generally at the nanometer scale, while their length can reach tens of nanometers or micrometers; thus, they are considered protein nanowires. In barnacle cement proteins, such as Mrcp-100k protein, self-assembly into amyloid fibers in seawater solutions is observed [5,62,63]. These fibers are highly stable in seawater, do not disassemble, and possess strong adhesiveness. The formation of such amyloid fibers is closely related to the curing process of barnacle glue, indicating their potential key role in the underwater adhesion of barnacles.

The self-assembly mechanism of non-amyloid nanofibers in barnacle cement proteins mainly involves interactions between protein molecules, including but not limited to π-π stacking [64], hydrogen bond formation, and hydrophobic interactions. Barnacle cement proteins, through fusion with elastin-like proteins (ELPs), form composite protein adhesives with self-assembly properties. These composite protein adhesives can rapidly self-assemble into nanofibers under specific conditions, such as temperature and concentration [65]. Non-amyloid nanofibers are formed under different conditions; for example, rBalcp19k proteins can self-assemble into non-amyloid nanofibers under acidic, low ionic strength conditions [30]. These nanofibers also exhibit high stability, remaining insoluble in seawater and resisting the adverse effects of high pH and high ionic strength. Furthermore, mutants lacking intramolecular disulfide bonds show a broader range of conditions for forming nanofibers, indicating that the presence or absence of disulfide bonds significantly affects the self-assembly of barnacle cement proteins into nanofibers.

In summary, both amyloid and non-amyloid nanofibers formed by the self-assembly of barnacle cement proteins demonstrate high structural stability and environmental adaptability. Amyloid nanofibers primarily form through cross-β structures, while non-amyloid nanofibers may form through different self-assembly mechanisms. These characteristics give them potential application value in the design of barnacle biomimetic medical materials.

### 2.3. The Adhesion Mechanism and Solidification Mechanism of Barnacle Cement Proteins

**Figure 1:**
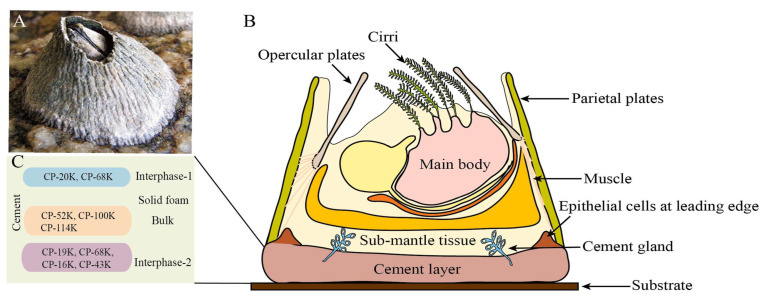
(**A**): Barnacle adhering to substrate by cement; (**B**): simplified schematic of adult acorn barnacle cross-section; (**C**): proposed protein-based molecular mechanism of barnacle cement permanent adhesion [69].

As a marine sessile organism, barnacles secrete a multi-protein complex known as barnacle cement, which enables strong attachment to various substrates in the ocean. Research on the adhesion mechanisms of barnacle proteins and the curing mechanisms of barnacle cement has always been a hot topic in the study of barnacle cement proteins. This research is not only of great significance for the control of marine biofouling but also provides new insights for the development of biomimetic materials based on barnacle cement proteins [66,67,68]. The structures related to barnacles can be seen in (Figure 1) [69].

The adhesion process of barnacle cement proteins involves a series of complex biological processes, mainly including substrate detection, signal transduction, cement secretion, and cement solidification [66]. As shown in Table 2, during the substrate detection phase, barnacles use the antennal sucker action of their nauplius larval stage to detect and selectively attach to suitable substrates through purely mechanical means [17]. Subsequently, through signal transduction mechanisms, barnacles activate the expression of genes related to cement secretion [5,70,71]. Then, barnacles secrete cement fluid containing various proteins through a network system of secretory glands connected by ducts, which is a key step in ensuring the even distribution of barnacle cement proteins and effective adhesion [72]. Moreover, to enhance adhesion, the protein complex SIPC secreted by barnacles plays an important role in the process of nauplius larvae selecting the adhesion substrate [33,73,74]. The proteins in barnacle cement fluid include but are not limited to cp19k and cp20k, which play complex and crucial roles in the underwater environment, allowing barnacles to firmly attach to various underwater substrates. For example, cp19k and cp20k proteins are responsible for adhesion to external substrates and the calcareous base of barnacles [22], while cp52k and cp100k proteins are responsible for higher-level interactions. These interactions not only involve direct contact between proteins, but may also involve deeper biochemical mechanisms, such as enzymatic reactions or ligand–receptor system actions [4,75]. Table 2 presents the four processes of barnacle adhesion on a substrate in seawater.

The curing mechanism of barnacle cement is the foundation for the long-term maintenance of its adhesive properties, involving multiple complex stages such as matrix clearance, cross-linking formation, and ultimate stability enhancement [76]. Studies have found that the matrix in barnacle cement first cleans the surface of the adhesive substrate material by repelling water and contaminants [10,77]. Subsequently, adhesion proteins form cross-links with the tissue surface, generating strong adhesive forces to achieve solidification [2,43]. In addition, the curing mechanism of barnacle cement is closely related to the self-assembly process of proteins in the cement fluid. Research indicates that the cp19k protein of the white ridge barnacle can self-assemble into amyloid fibrils in seawater solutions, demonstrating the efficient underwater adhesion capability of barnacle cement [78]. Molecular recognition is a key mechanism in the formation of barnacle amyloid adhesives; these findings provide significant insights into the formation of barnacle cement and offer ideas for the design of new underwater adhesives [79].

The curing of barnacle cement likely involves a multitude of chemical and enzymatic reactions. For instance, enzymatic components within the cement may facilitate crosslinking and the solidification of the adhesive fluid through catalytic reactions, thereby enhancing its adhesive strength [80]. SIPC, a macromolecular signaling protein predominantly distributed in the barnacle’s viscera, shell, and larval footprints, exerts its function through direct contact with the antennae of barnacle larvae. Its inducing activity may be attributed to different structural configurations or glycosylation modifications [81], although the specific mechanisms of action remain yet to be fully elucidated [71]. Studies have shown that SIPC may activate ion channels by binding to G protein-coupled receptor (GPCR), inducing the influx of calcium ions and stimulating the secretion of vesicles from the endoplasmic reticulum, thereby participating in the settlement process of cyprids [82]. Additionally, enzymatic components within barnacle cement, such as lysyl oxidase and peroxidase, have demonstrated homology with silk fibroin and may play a crucial role in the curing process of barnacle cement [83]. The key proteins such as SIPC in barnacle cement, along with various enzymes and enzyme inhibitors, play an essential role in stable attachment through their involvement in signal transduction, cement curing, and structural stabilization processes. These discoveries not only update our understanding of barnacle cement but also provide new avenues for the development of biomimetic underwater adhesive materials [80].

Although there is a certain understanding of the adhesion mechanisms and curing mechanisms of barnacle cement proteins, many mysteries remain unsolved. For example, the specific molecular structure of barnacle cement proteins, their self-assembly behavior, and interactions with other biomolecules still require further research [67]. Moreover, how to mimic the adhesion and curing processes of barnacle cement proteins and how to apply these mechanisms to the development of biomimetic underwater adhesives are also current research focuses and challenges [84].

Through the study of the adhesion mechanisms and curing mechanisms of barnacle cement, we can not only gain a deep understanding of how marine organisms achieve firm underwater attachment but also provide a theoretical basis and technical support for the development of new adhesives in moist environments. Future research needs to further reveal the specific molecular mechanisms of barnacle cement proteins and explore the potential of barnacle biomimetic materials in the biomedical field, including efficient wet adhesion, multifunctionality, biocompatibility and degradability, environmental friendliness, and the feasibility of engineered production.

### 2.4. The Influence of pH on the Self-Assembly Behavior and Adhesive Properties of Barnacle Cement Proteins

The structural characteristics of barnacle cement also include its varying responsiveness to environmental factors. This section primarily discusses how the expression and function of barnacle cement proteins are influenced by pH levels, with different pH values affecting protein self-assembly behavior and adhesive properties, similar to the self-assembly behavior of various proteins and peptides.

**Figure 2:**
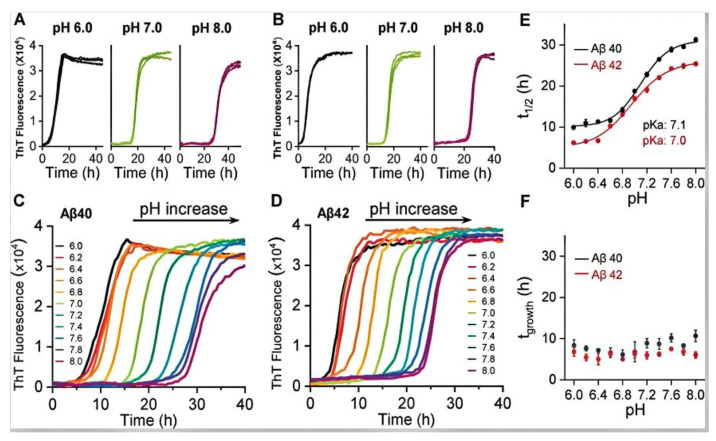
The pH-dependent fibril formation kinetics of Aβ40 and Aβ42. ThT kinetic traces (n = 4) at pH 6.0, 7.0, and 8.0 for Aβ40 (**A**) and Aβ42 (**B**); see also supplementary S2 and S3. Single representative traces for Aβ40 (**C**) and Aβ42 (**D**) between pH 6.0–8.0, from left (black, pH 6.0) to right (purple, pH 8.0). (**E**) Plots of t 1/2 versus pH, with pK a fitted. (**F**) Plots of growth-time versus pH; error bars are the standard error of the mean (SEM) from four replicates [91].

Different pH values can impact the self-assembly behavior of barnacle cement proteins in terms of structure, rate, and stability. According to research [85], barnacle cement proteins can form different self-assembled structures under various pH conditions. For instance, at pH levels of 2–5, positively charged left-handed helical nanotubes may form; while at pH levels of 6–8, negatively charged right-handed helical nanoribbons may form. Additionally, changes in pH can affect the morphology of self-assembled structures, such as transitions from nanotubes to nanoribbons, or from flat ribbon-like structures to twisted right-handed structures [86]. Variations in pH also influence the self-assembly rate of barnacle cement proteins. Under neutral pH conditions, some proteins may self-assemble at a slower rate [87], while under acidic or alkaline conditions, the self-assembly rate may accelerate [88]. Different pH levels can also affect the stability of self-assembled structures. Under acidic conditions (lower pH values), some proteins may form more stable fibrous structures, while under alkaline conditions (higher pH values), different nanostructures may form or remain as monomers [89,90]. Particularly around a pH of 7, some proteins exhibit better self-assembly capabilities, forming nanofibers with specific microstructures [87,88]. This is shown in (Figure 2) and in [91].

The adhesive properties of barnacle cement proteins under different pH conditions are also affected accordingly. Under acidic conditions, as the pH increases, the adhesiveness of some proteins is enhanced; while under alkaline conditions, their adhesiveness may diminish [30]. This variation is likely related to the secondary structure transitions and self-assembly capabilities of the proteins. For example, nanofibers formed in acidic environments may provide a stronger adhesive interface for barnacle cement [30,92]. Additionally, changes in pH can affect the physical properties of proteins, such as gel strength and thermal stability, thereby indirectly influencing their adhesive characteristics.

In summary, different pH levels can affect the charge state of barnacle cement proteins, the morphology and stability of self-assembled structures, and the rate of self-assembly, thereby significantly impacting their self-assembly behavior and adhesive properties. By adjusting the pH, the spatial structure of barnacle cement proteins can be altered, effectively regulating the self-assembly behavior and the strength of adhesive forces. The pH sensitivity of barnacle cement proteins holds great research and application potential in the field of biomimetic materials for biomedical applications.

### 2.5. The Differential Adsorption of Barnacle Cement on Various Substrate Materials

Barnacle cement, primarily composed of protein subunits, shares a similar basic composition between primary and secondary cements [93]. This structure endows barnacle cement with certain adhesiveness and adaptability, allowing it to form effective adhesion with different substrate material surfaces. However, the adsorption and adhesion behavior of barnacle cement on different substrate materials exhibit variations, a phenomenon that can be explained from multiple perspectives.

The adhesive performance of barnacle cement is significantly influenced by the surface properties of the substrate material. For instance, the porosity, surface permeability, and pH variations in the substrate can all affect the attachment behavior of barnacles [94]. Moreover, surfaces with micro-hook structures, such as the hooks of Galium aparine (cleavers), can enhance dry adhesion [95]. Homologous proteins of approximately 19kDa in both the white ridge barnacle and the red giant barnacle play a crucial role in surface attachment, yet their isoelectric points differ significantly, at 10.3 and 5.8, respectively, leading to differences in their interactions with S_i_O_2_ surfaces [96]. The interaction between barnacle cement and substrate materials also involves intermolecular forces, such as van der Waals forces, hydrogen bonds, and ionic bonds. These interactions help to enhance adhesive strength, especially when the substrate surface possesses specific chemical groups. Additionally, the surface tension of the substrate material can affect the adhesion force of barnacle cement [97].

The adhesion of barnacle cement is also related to the interactions of its internal components. It has been reported that the adsorption force and amount of Mrcp-19k on SiO_2_ surfaces are less than those of Bacp-19k [96]. This indicates that different protein components exhibit varying adhesion performance on different substrate materials. This suggests that the use of different compositional components in barnacle biomimetic adhesives, such as additives, can also affect their interactions with substrate materials. Moreover, the curing mechanism of barnacle cement is also a key factor affecting its adhesion. The analysis of pattern barnacle cement proteins and the study of their curing mechanism show that the process of cement protein self-assembly into amyloid fibrils is closely related to the curing of barnacle cement [78]. This implies that the curing process of barnacle cement on different substrate materials may be influenced by the characteristics of the substrate material, thereby affecting its final adhesion performance.

The interaction between barnacle cement and substrate materials is also affected by environmental conditions such as temperature, humidity, and pH values. For example, when the adhesive is exposed to an acidic environment, its mechanical properties may decrease. Additionally, storage conditions (such as dryness, water, or lactic acid) can affect the surface roughness of the adhesive and the ultimate tensile strength [98].

In summary, the responsiveness of barnacle cement to the environment enables it to rapidly cure and form strong adhesion in underwater environments, providing a solid attachment foundation for barnacles. The differences in adhesion of barnacle cement on different substrate materials result from the combined effects of substrate material surface characteristics, interactions of internal components of barnacle cement, curing mechanisms, and environmental conditions. These differences are significant for understanding the adhesion mechanism of barnacle cement and for the development of new types of barnacle biomimetic adhesives and other biomedical materials.

## 3. The Development of Barnacle Biomimetic Materials

### 3.1. Status Analysis

Based on the unique adhesion and curing mechanisms of barnacle cement, as well as its biological properties, barnacle biomimetic medical materials have shown significant potential for development, with the expectation of creating new medical materials with excellent adhesion and good biocompatibility. Concurrently, biomimetic technology in the biomedical field is continuously expanding. For instance, biomimetic medical adhesives are gradually becoming a research hotspot due to their diverse forms, high tissue adhesion strength, superior mechanical properties, and improved biocompatibility [99].

Barnacle biomimetic materials possess unique advantages in the biomedical field, such as good biocompatibility and antimicrobial properties. However, they also face limitations such as complex preparation and the need for validation of long-term stability, and further research and verification are required regarding their stability and biodegradability within the body [44,100]. The aim of this section is to review the latest research developments in barnacle biomimetic medical materials, providing relevant information and suggestions for subsequent research on barnacle biomimetic materials to promote the advancement of this field.

#### 3.1.1. High-Strength Medical Adhesives

**Figure 3:**
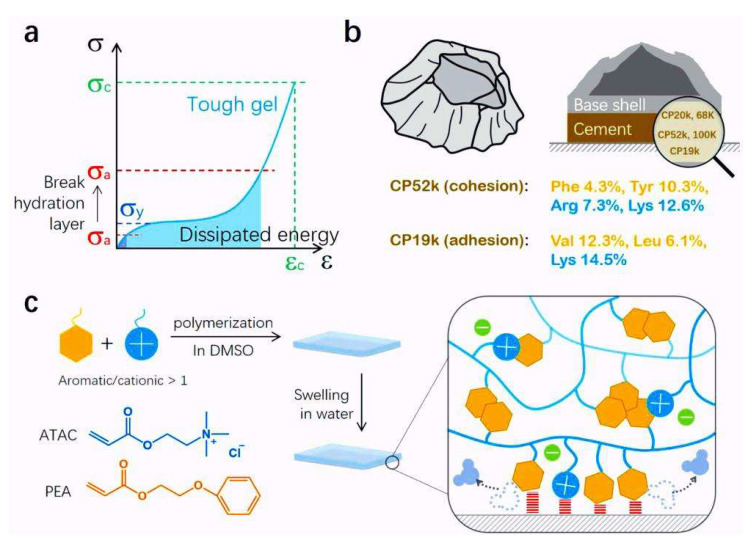
The schematic design strategy for hydrogel adhesives with robust, long-lasting, and repeatable underwater adhesion. (**a**) An illustration of the general mechanical principle for a strong adhesive. The blue curve represents the bulk tensile stress–strain curve of a soft, but tough, material with yielding stress (σ_y_), fracture stress (σ_c_), and fracture strain (ε_c_). The red dashed lines represent the debonding stress (σ_a_) of the adhesive interface. A strong but reversible adhesive requires a large σ_c_ and ε_c_ in bulk and a large σ_a_ at the interface with σ_y_ << σ_a_ < σ_c_, allowing for significant energy dissipation without cohesive failure. For hydrogel adhesives, while large σ_c_ and ε_c_ can be obtained by dynamic bonds, achieving a large σ_a_ in water is challenging due to the hydration of interfaces underwater. (**b**) A schematic of a barnacle, its cross-section, and the cationic (Arg and Lys) and hydrophobic (Phe, Tyr, Val, and Leu) amino acid contents in the two CPs: CP52k for cohesion in the bulk and CP19k for adhesion at the interface. (**c**) A schematic of the molecular mechanism to fabricate underwater adhesive hydrogels. Cationic 2-(acryloyloxy)ethyl trimethylammonium chloride (ATAC) and aromatic 2-phenoxyethyl acrylate (PEA) monomers were adopted to mimic the cationic and hydrophobic amino acids in barnacle CPs, respectively. π–π and cation–π interactions provide dynamic bonds in the bulk hydrogel to show high toughness, while they cause dehydration at the interface to promote electric and hydrophobic interactions, which results in a large σ_a_ [102].

In recent years, the research boom in biomimicry has propelled the rapid development of material science and the biomedical field, with significant progress achieved in the application of barnacle biomimetic materials in medical adhesives. The adhesive proteins in barnacle cement, such as cp19k, cp20k, cp52k, and cp100k, play a central role in the underwater adhesion process. These proteins achieve a strong adhesion to the substrate surface through various interactions, including physical adsorption, coordination bonds, enzymatic reactions, and hydrophobic forces [68]. Professor Zhao Xuanheo’s team at MIT has developed an emergency hemostatic bio-adhesive inspired by barnacle glue that can form a strong adhesion within 15 s and is independent of blood coagulation [10]. This bio-adhesive, composed of a hydrophobic oil matrix and bio-adhesive particles, mimics the dual function of high-density lipids and adhesive proteins found in barnacle cement, providing an effective tool for wound closure. Furthermore, Professor Hu Biru’s biomimetic biology team at the National University of Defense Technology has developed a biomimetic adhesive with strong underwater adhesion performance by delving into the attachment mechanisms of barnacles [101]. By combining the attachment mechanisms of barnacles with modern bioengineering technology, such barnacle biomimetic adhesives can function well in moist environments, potentially offering a new approach for the surface modification of drug-coated implants within the human body. The adhesive properties of barnacle cement proteins make them an ideal raw material for medical adhesives. Researchers such as Fan et al. [102], through an in-depth investigation of the amino acid composition of barnacle cement proteins, have successfully developed a barnacle biomimetic hydrogel with excellent wet adhesion properties. The team’s hydrogel design scheme is shown in (Figure 3) [102].

**Figure 4:**
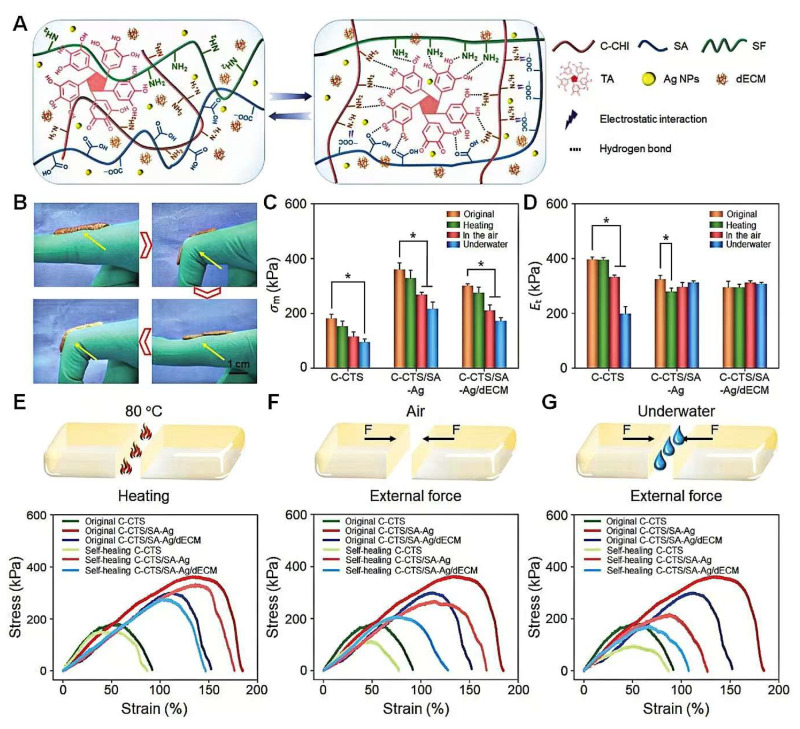
The self-healing properties of the hydrogels. (**A**) A schematic illustration of the self-healed hydrogel structures. (**B**) Macroscopic observation showing the self-healing course of the hydrogels adhered to a finger. (**C**) Tensile strength and (**D**) Young’s modulus comparisons of the original and self-healed hydrogels. Stress–strain curves of the original and self-healed hydrogels (**E**) with heating; (**F**) in air; and (**G**) under water. All statistical data are represented as mean ± SD (n = 3; * *p* < 0.05) [103].

Inspired by barnacles, Professor Yang Huanghao’s team at Fuzhou University first proposed a mussel–barnacle cement protein-inspired double biomimetic hydrogel [103]. As shown in (Figure 4) [103], the dual-bionic bio-adhesive designed by Professor Yang Huanghao’s team at Fuzhou University still maintains its high adhesive strength even after multiple bonding and debonding cycles, as well as prolonged soaking. Due to its strong tissue adhesion strength and the hydrogel’s outstanding self-healing behavior, skin incisions can automatically contract through mechanical forces during the wound-healing process, providing a new strategy for wound closure without surgical sutures. Researchers have prepared barnacle cement proteins into biomedical adhesives with high strength and lasting adhesion through methods [2] such as chemical crosslinking and physical curing. These adhesives can maintain stable adhesion in wet environments and are suitable for various surgical procedures and trauma repairs. Compared to traditional suturing methods, barnacle biomimetic medical adhesives offer the advantages of simplicity, faster healing, and less scarring, providing new options for clinical surgery.

Due to the intricate internal environment within biological organisms, which differs from the natural habitat of barnacles, it may lead to alterations in the structure and performance of the organisms over time. The complex and variable internal environment of the human body can affect the stability of barnacle glue biomimetic materials within it, influenced by various factors such as immune system recognition and attack. The rate of degradation of barnacle biomimetic materials within the biological body should be commensurate with the rate of tissue integration. Excessive degradation may lead to structural instability, while insufficient degradation could hinder the formation of functional tissue. Therefore, when researching and developing new barnacle biomimetic materials, it is essential to consider the impact of environmental factors on the degradation of these new materials, including changes in pH and enzymatic activity. Additionally, researchers must ensure that the small molecules produced by the degradation of biomaterials are biocompatible and do not cause inflammatory responses or toxicity. Hence, toxicological studies are required to assess the safety of the degradation products of barnacle glue biomimetic materials. Over time, barnacle glue biomimetic materials may undergo aging and degradation processes, leading to a decline in their performance. Such degradation may be caused by factors such as oxidative stress, enzymatic action, or microbial invasion. These complex changes can affect the mechanical strength, adhesion, and durability of barnacle biomimetic materials. To assess the long-term stability of barnacle glue proteins, long-term clinical follow-up studies are necessary, which increase the research and development cycle and costs.

#### 3.1.2. Tissue Engineering

The application of barnacle biomimetic materials in tissue engineering is one of the hot topics in current research. Barnacle cement not only possesses excellent biocompatibility and self-assembly properties but also promotes cell adhesion and proliferation, making it an ideal material for tissue engineering. Researchers have leveraged the self-assembly characteristics of barnacle cement proteins to develop a series of novel biomedical materials, such as functional hydrogels and films. Barnacle biomimetic polypeptide hydrogels have a strong tissue repair capability and can effectively promote cell growth and tissue remodeling processes, making them particularly suitable for the repair of skin and soft tissue injuries in the human body [104]. The introduction of this material has made a qualitative leap in traditional tissue repair and regeneration methods, opening up new avenues for disease treatment. For instance, Fujii and colleagues utilized the self-assembly properties of the adhesive protein cp52k to design polypeptide hydrogels as candidate materials for tissue engineering scaffolds [105]. Inspired by the adhesion performance of marine barnacles, Professor Zhao Xia’s team from the School of Medicine and Ocean Drug Ministry Key Laboratory at Ocean University of China, based on chitosan (CTS) and 2-phenoxyethyl acrylate (PEA), developed a functional chitosan-based composite hydrogel with strong wet adhesion properties [106]. The combination of chitosan and barnacle cement proteins significantly enhances the mechanical performance and biocompatibility of the material, providing strong support for biological tissue engineering.

Given the complexity of the internal human environment, simulating the intricate microenvironment within the body and enhancing the mechanical strength and durability of biomimetic scaffolds represent current challenges. To address these issues, this review proposes several potential solutions. Researchers can employ multifactorial dynamic culture systems, three-dimensional printing technology, bioreactor technology, co-culture systems, computer simulations, and tissue engineering to simulate the complex microenvironment within the body. This review has listed the design ideas and possible solutions for these aspects in Table 3.

In terms of enhancing the mechanical strength and durability of biomaterials, suitable barnacle biomimetic materials can be designed through the following approaches: (1) selecting biocompatible materials with superior mechanical properties, such as composites of polymers, proteins, and inorganic materials, to optimize the mechanical strength of the scaffold through material design; (2) modifying the micro- and macrostructure of the scaffold, such as increasing cross-linkages, adjusting porosity, and designing specific geometric shapes, to enhance its mechanical strength and durability; (3) chemically or physically modifying the scaffold surface to enhance its wear resistance, fatigue resistance, and degradation resistance; and (4) utilizing nanotechnology to enhance the mechanical properties of the scaffold, such as doping with nanoparticles, nanoscale surface modification, or constructing nanocomposite materials. Subsequently, researchers must conduct long-term in vitro and in vivo testing of the designed materials to assess the durability of the scaffold in a simulated in vivo environment and iterate optimization based on test results. Concurrently, the degradation characteristics of barnacle glue biomimetic materials in tissue engineering applications must be synchronized with the processes of cell growth and tissue reconstruction, allowing the biomaterial to promote tissue regeneration while gradually degrading. In summary, simulating the complex microenvironment within the body and enhancing the mechanical strength and durability of scaffolds require interdisciplinary research and innovation to develop more effective and safer biomaterials for clinical needs.

Biomimetic mineralized collagen materials, often used in the form of barrier membranes in guided bone regeneration surgery [14,118], are gradually replacing traditional non-absorbable membranes due to their good bio-absorbed ability and fewer postoperative complications [119,120]. The mineralization properties of barnacle glue protein endow it with potential applications in bone tissue engineering, such as serving as a barrier membrane for bone repair. Huang and colleagues [75], by mimicking the structure and function of cp20k protein, designed a series of biomimetic peptides with biomineralization capabilities. These biomimetic peptides can induce the deposition and growth of minerals both in vitro and in vivo, providing new ideas and methods for bone tissue engineering. However, when applying these materials to clinical bone repair, there are certain limitations and challenges, particularly in terms of matching the degradation rate of the materials with the growth rate of newly formed bone tissue. It is well understood that the premature degradation or excessively slow degradation of biomaterials can adversely affect bone repair outcomes. Barnacle biomimetic materials may elicit host-immune or inflammatory responses in practical applications, which can compromise the stability and efficacy of the materials. The long-term stability and durability of barnacle biomimetic materials within the body are not yet fully established, particularly under conditions of repetitive loading. Furthermore, if the mechanical properties of the designed biomaterials do not match those of the bone tissue, this could lead to stress concentration or stress shielding, thereby affecting the repair of bone tissue. Researchers can enhance the efficacy of barnacle biomimetic materials in bone repair by investigating strategies to improve the congruence between the degradation rate of the materials and the growth rate of neo-bone tissue.

Barnacle cement, known for its strong adhesion under wet conditions and non-toxic properties, has been a subject of research for dental restorative applications [121,122]. The enamel mineralization pathway refers to the process in which minerals, such as calcium and phosphorus, are deposited to form a hard structure in teeth or similar structures. Gene Ontology enrichment analysis shows that differentially expressed genes are significantly enriched in the enamel mineralization pathway [82], indicating that SIPC is related to the calcification process of barnacle cyprids, promoting the growth of the basal plate, which is a key step in the formation of organic matrices such as barnacle shells and enamel. The discovery of this mechanism provides potential molecular targets for the development of new curing materials and helps in designing new schemes for tooth repair.

#### 3.1.3. Drug Delivery Systems

The development of drug delivery systems has always been a challenge in the pharmaceutical industry. Barnacles, due to their secretion of special mucus structures, have inspired the creation of hydrogels with adjustable release functions, which have garnered significant attention. The self-assembly characteristics of barnacle cement proteins enable them to form stable nanostructures, providing an excellent carrier for drug loading and release [16]. Biomimetic peptides based on barnacle cement proteins exhibit excellent gelation capabilities, allowing for the formation of hydrogels with short gelation times, higher storage modulus, and greater mechanical strength even at low peptide concentrations. These self-assembled peptide hydrogels possess good biocompatibility and can be widely applied in biomedical fields such as drug release and cell culture. Researchers such as Hu et al. have proposed the application of barnacle cement proteins or their derived peptides in the design of drug delivery systems [123]. By employing chemical modification and physical encapsulation methods, researchers have combined drugs with barnacle cement proteins to prepare drug delivery systems with targeting and controlled release properties [16,124,125]. These systems are capable of releasing drugs at specific times and sites, enhancing the bioavailability and therapeutic efficacy of drugs while reducing side effects. Barnacle biomimetic materials also exhibit good biodegradability, safely degrading within the body and avoiding the risks associated with secondary surgeries.

Barnacle glue biomaterials require further optimization of drug loading efficiency and release kinetics in drug delivery systems to achieve more precise dose control. Researchers can consider designing drug loading efficiency from multiple aspects, such as utilizing molecular design by introducing hydrophilic or hydrophobic groups to improve drug compatibility with the carrier, or employing nanotechnology, such as nanoparticles and nano capsules. Biomaterial developers need to design controlled release systems based on specific mechanisms, such as diffusion control, degradation control, pH sensitivity, temperature sensitivity, and enzyme sensitivity, to achieve timed and quantified drug release to match the needs of drug delivery. To evaluate the application of barnacle biomimetic materials in drug delivery systems, in vitro and in vivo release tests are necessary, such as in vitro release kinetics experiments and animal model tests, to assess and optimize drug release behavior. Based on the test results, adjust the composition and structure of the carrier material to optimize the drug release curve. Subsequently, combining pharmacokinetic and pharmacodynamic data, conduct rigorous extensive clinical trials to evaluate the actual effect of the drug delivery system and make adjustments as needed to optimize the drug release plan. This not only improves therapeutic outcomes but also reduces the toxic side effects of drugs, enhancing the quality of life for patients.

#### 3.1.4. Antimicrobial Medical Materials

To prevent fatal issues such as bacterial infections and thrombosis, the functional modification of biomaterials surfaces is necessary. Leveraging the excellent properties of barnacle cement, including its antibacterial and degradable characteristics, barnacle cement proteins are also used as bio-crosslinking agents. Combined with chemical synthesis methods, a multifunctional polymer drug coating with anti-protein adsorption as well as antibacterial and anti-biofouling properties has been prepared [8,126], playing an active role in the field of biomaterials’ surface property regulation. The multifunctional coating containing barnacle cement proteins exhibits good cell compatibility, and is capable of suppressing inflammatory responses and promoting the M2 polarization phenotype of macrophages [8].

The specific mechanisms of action and efficacy of the antimicrobial components in barnacle cement biomaterials are two complex and critical areas that require in-depth research to ensure their safety and effectiveness. For the development and clinical translational research of barnacle glue biomimetic antimicrobial materials, clinical trials can be conducted to assess the long-term clinical outcomes of barnacle glue proteins, including infection rates, healing times, recurrence rates, and patient satisfaction. However, current biological experiments on barnacle biomimetic materials are insufficient, and more comprehensive research is needed to ensure the safety and effectiveness of the antimicrobial components within barnacle biomimetic materials.

### 3.2. Trend Forecasting

Barnacle biomimetic materials are anticipated to see continuous expansion in their applications within the fields of tissue engineering and drug delivery systems, owing to their exceptional biocompatibility and self-assembly properties [16,127]. As scientific and technological advancements deepen our understanding of the self-assembly characteristics of barnacle cement proteins, it is foreseeable that a variety of novel biomedical materials, such as hydrogels and films [102], will be developed. These materials not only promote cell adhesion and proliferation but also provide a stable carrier for drug loading and release. The application of barnacle biomimetic materials in ocular drug delivery systems is also garnering significant attention [61]. With the progress and the development of science and technology over time, modern biomimetic materials require appropriate functional features to meet the needs of human societal development. This section will discuss the following three aspects.

#### 3.2.1. Further Functionalization of Barnacle Biomimetic Materials

In the future, research on barnacle biomimetic materials will place greater emphasis on the functionalization of these materials. Barnacle cement proteins possess strong adhesion and anti-flushing characteristics, making them ideal for the development of high-performance biomimetic adhesives. Consequently, future efforts will focus on enabling barnacle cement proteins not only to adhere to inorganic and organic surfaces but also to form irreversible covalent bonds through oxidation processes, thereby enhancing adhesion strength [128]. Through chemical modifications and genetic engineering, researchers can endow barnacle biomimetic materials with additional functional properties, such as antimicrobial, anti-inflammatory, and angiogenesis-promoting capabilities. Moreover, in the study of barnacle biomimetic materials, it is crucial for biomimetic medical materials to exhibit good biocompatibility, antibacterial properties, and degradability, which are particularly important in the field of biomedicine. Continuously improving the functional characteristics of barnacle biomimetic materials can enhance their application potential and value in the field of biomedicine, meeting the growing clinical demands.

Furthermore, research on biomimetic peptides based on barnacle bio-adhesive proteins, self-assembled hydrogels, and their preparation processes and applications has demonstrated the potential of barnacle cement in the field of smart materials, such as self-healing materials and sensor design.

To overcome the limitations of barnacle glue biomimetic materials, researchers can improve the stability and biodegradability of barnacle cement proteins through chemical modification or physical treatment to meet various clinical needs. Concurrently, researchers should develop reliable in vitro and in vivo models to simulate the behavior of barnacle cement proteins within the human body, predicting their long-term stability and degradation characteristics. Rigorous biological experiments and clinical trials should be conducted to assess the performance of multifunctional barnacle biomimetic materials.

#### 3.2.2. Multiscale Design and Fabrication of Barnacle Biomimetic Materials

Currently, the extraction and purification processes of barnacle cement proteins may be relatively complex, increasing production costs. This implies that the complexity in the preparation of barnacle biomimetic materials urgently needs to be addressed, requiring researchers to design suitable new materials. With the continuous advancement of nanotechnology and biotechnology, the multiscale design and fabrication of barnacle biomimetic materials will become a focal point of future research. Through multiscale design, researchers can precisely control the structure and properties of barnacle biomimetic materials, achieving optimization at both the micro- and macroscales. To address the drug release challenges of barnacle biomimetic medical materials in complex microenvironments, future research needs to focus on optimizing the design of drug carriers, such as enhancing drug loading capacity and achieving sustained drug release capabilities, to maximize their therapeutic effects. This will aid in the development of barnacle biomimetic materials with higher performance and application value. Inspired by the barnacle’s use of amyloid-like protein systems to form stable aqueous adhesion on solid surfaces, wet material surfaces can be modified with protein nanofilms, thereby promoting rapid multiple interactions between the protein nanofilms and the wet surfaces [129].

#### 3.2.3. The Clinical Translation and Industrialization of Barnacle Biomimetic Materials

The clinical translation and industrialization of barnacle biomimetic materials will be an important direction for future development. By employing genetic engineering techniques to synthesize recombinant proteins, among other methods, the performance of barnacle cement can be further optimized, expanding the application range of barnacle-inspired biomaterials and promoting their industrial development. However, the commercialization process of barnacle biomimetic medical materials faces numerous challenges, including regulatory requirements, high costs and complexity in manufacturing, market size issues, and obstacles related to storage and distribution. Researchers need to strengthen collaboration with clinical physicians and enterprises to clarify product advantages, improve product deficiencies, and jointly promote the clinical application and industrialization process of barnacle biomimetic materials. Through clinical trials and market promotion, barnacle biomimetic materials have the potential to become significant innovative products in the field of biomedicine, contributing to the cause of human health.

Currently, as a novel biomaterial, barnacle cement proteins may elicit immune responses or toxicity, necessitating extensive biological safety assessments. Furthermore, after the design and development of barnacle biomimetic materials, their long-term stability and biodegradability in the body still require long-term research and clinical trials for validation. Additionally, maintaining the consistency and reproducibility of material properties during the transition from laboratory research to industrial production is also a challenge. Moreover, the commercialization of medical materials must meet stringent regulatory and supervisory requirements, which may delay the product’s market launch. Overall, although the adhesion and curing mechanisms of barnacle cement offer unique advantages for the development of new biomaterials, various technical and clinical challenges still need to be overcome for practical applications. Future research needs to delve into material design, production technology, safety assessment, and clinical trials to achieve the widespread application of these materials.

## 4. Conclusions and Outlook

This article provides a detailed review of the latest research advancements of barnacle cement and its biomimetic materials in the field of biomedicine, with a focus on exploring the fundamental properties, adhesion mechanisms, and diverse applications of barnacle cement in biomedicine. It delves into the potential applications of barnacle cement and its biomimetic materials within the biomedical domain. Additionally, the article elucidates the scientific principles underlying the efficient underwater adhesion of barnacle cement proteins. Based on the adhesion properties of barnacle glue, researchers have developed a series of new medical materials, providing innovative solutions for surgery, wound repair, and drug delivery.

In the future, research will continue to delve into the adhesive mechanism of barnacle cement proteins and explore their novel applications in the field of biomedicine. It is anticipated that barnacle-inspired materials will see a sustained expansion in applications such as tissue engineering and drug delivery systems. These materials not only promote cell adhesion and proliferation but also provide a stable vehicle for drug loading and release. Furthermore, with advancements in nanotechnology and biotechnology, the structure and properties of barnacle-inspired materials are expected to be further optimized to meet a broader range of clinical needs. In terms of material functionalization, future studies will focus on endowing barnacle-inspired materials with additional functional properties, such as antimicrobial, anti-inflammatory, and pro-angiogenic capabilities, through chemical modification and genetic engineering, to address the growing demands in clinical settings. Concurrently, the development of multi-scale design and manufacturing technologies will enable researchers to more precisely control the structure and properties of barnacle-inspired materials, achieving optimization at both the micro- and macroscales.

Overall, barnacle gum and its biomimetic materials have broad application prospects in the field of biomedicine. Through an in-depth study of its adhesion mechanism and properties, the development of barnacle biomimetic materials with more functional characteristics, and continuous innovation of its preparation technology and application methods will promote the clinical transformation and industrialization of barnacle biomimetic medical materials. It is expected to become an important innovative product in the field of biomedicine, make greater contributions to human health, and bring more innovations and breakthroughs to the field of biomedicine.

## Figures and Tables

**Table 1:** Summary of different analytical methods.

Experimental Methods	Analytical Approaches
IR	Collagen molecules undergo spontaneous self-assembly to form native collagen fibrils. Fourier Transform Infrared Spectroscopy (FTIR) can be used to monitor the heat-induced fibril formation process in aqueous media in real-time.
UV–Vis	Based on the measurement of protein absorption in the ultraviolet and visible light regions, molecular events during protein self-assembly can be inferred, and the rate of self-assembly can be studied.
TM	Measure the changes in solution turbidity, thereby indirectly reflecting the extent of protein self-assembly.
FS	Select appropriate fluorescent molecules as probes to interact with proteins and provide information about changes in the protein environment, thereby inferring the behavior of protein self-assembly.
CD	Based on the difference in absorption of left-handed and right-handed circularly polarized light by substances, detect the changes in the secondary structure of proteins during the self-assembly process.
NMR	Through methods such as three-dimensional structure determination, probing conformational changes, monitoring the self-assembly process, and identifying self-assembled products.
SEM	By comparing SEM images at different time points and analyzing morphological characteristics, infer the molecular interactions and assembly mechanisms during the protein self-assembly process.
AFM	By detecting the interactive forces between the sample surface and the probe, it is possible to directly observe the morphology, structure, and dynamic behavior of protein self-assemblies, as well as measure their mechanical properties.
TEM	Directly observing the morphology and structure of protein self-assemblies can also be used to verify the self-assembly conditions obtained through protein crystallization screening methods.
CLSM	Through fluorescent labeling and high-resolution imaging techniques, it is possible to precisely observe and analyze the morphology, structure, and dynamic processes of protein self-assemblies.

**Table 2:** Barnacle adhesion process.

Phase	Event	References
Substrate detection	Barnacles use the action of the antennal suckers during the cyprid larval stage to detect and select suitable substrates for simple mechanical attachment.	[17]
Signal transduction	Through signal transduction mechanisms, barnacles activate the expression of genes related to cement secretion.	[5,70,71]
Cement secretion	The secretory gland connects to a duct network system that secretes a glue containing a variety of proteins, which is a key step in ensuring the even distribution of barnacle cement proteins and achieving effective adhesion.	[72]
Cement solidification	Adhesive proteins form cross-links with the tissue surface, generating strong adhesion, thereby achieving solidification.	[2,43]

**Table 3:** Simulation of in vivo microenvironment strategies.

Plan	Method	References
Multifactorial dynamic culture systems	Developing a multifactorial dynamic culture system to simulate the physical, chemical, and biological factors within the body, such as temperature variations, pH levels, oxygen gradients, extracellular matrix components, and the dynamic changes in biomolecules.	[107,108]
Three-dimensional printing technology	Utilizing three-dimensional printing technology to construct scaffolds with complex structures that mimic the architecture and function of in vivo tissues. These scaffolds can incorporate porous structures to facilitate cell infiltration and the transport of nutrients.	[109,110,111]
Bioreactor technology	Employing bioreactors to simulate mechanical stimuli within the body, such as pressure, tension, and vibration, which are crucial for cell behavior and tissue development.	[112,113]
Co-culture systems	Designing co-culture systems to cultivate different types of cells with biomaterials to simulate the interactions and signal transduction between different cell types within the body.	[114,115]
Computer simulations	Utilizing computer simulation technology to predict the behavior of materials within the body, and combining tissue engineering techniques to construct bioactive tissue models for drug screening and material testing.	[116,117]
